# Performance comparison of the Maxim and Sedia Limiting Antigen Avidity assays for HIV incidence surveillance

**DOI:** 10.1371/journal.pone.0220345

**Published:** 2019-07-26

**Authors:** Joseph B. Sempa, Alex Welte, Michael P. Busch, Jake Hall, Dylan Hampton, Shelley N. Facente, Sheila M. Keating, Kara Marson, Neil Parkin, Christopher D. Pilcher, Gary Murphy, Eduard Grebe, on behalf of the Consortium for the Evaluation and Performance of HIV Incidence Assays (CEPHIA)

**Affiliations:** 1 DST-NRF Centre of Excellence in Epidemiological Modelling and Analysis (SACEMA), Stellenbosch University, Stellenbosch, South Africa; 2 Vitalant Research Institute, San Francisco, CA, United States of America; 3 University of California San Francisco, San Francisco, CA, United States of America; 4 Public Health England, London, United Kingdom; 5 Facente Consulting, Richmond, CA, United States of America; 6 Data First Consulting, Belmont, CA, United States of America; University of Pretoria, SOUTH AFRICA

## Abstract

**Background:**

Two manufacturers, Maxim Biomedical and Sedia Biosciences Corporation, supply CDC-approved versions of the HIV-1 Limiting Antigen Avidity EIA (LAg) for detecting ‘recent’ HIV infection in cross-sectional incidence estimation. This study assesses and compares the performance of the two assays for incidence surveillance.

**Methods:**

We ran both assays on a panel of 2,500 well-characterized HIV-1-infected specimens. We analysed concordance of assay results, assessed reproducibility using repeat testing and estimated mean durations of recent infection (MDRIs) and false-recent rates (FRRs) for a range of normalized optical density (ODn) thresholds, alone and in combination with viral load thresholds. We defined three hypothetical surveillance scenarios, similar to the Kenyan and South African epidemics, and a concentrated epidemic. These scenarios allowed us to evaluate the precision of incidence estimates obtained by means of various recent infection testing algorithms (RITAs) based on each of the two assays.

**Results:**

The Maxim assay produced lower ODn values than the Sedia assay on average, largely as a result of higher calibrator readings (mean OD of 0.749 vs. 0.643), with correlation of normalized readings lower (*R*^2^ = 0.908 vs. *R*^2^ = 0.938). Reproducibility on blinded control specimens was slightly better for Maxim. The MDRI of a Maxim-based algorithm at the ‘standard’ threshold (ODn ≤1.5 & VL >1,000) was 201 days (95% CI: 180,223) and for Sedia 171 (152,191). The difference Differences in MDRI were estimated at 32.7 (22.9,42.8) and 30.9 days (21.7,40.7) for the two algorithms, respectively. Commensurately, the Maxim algorithm had a higher FRR in treatment-naive subjects (1.7% vs. 1.1%). The two assays produced similar precision of incidence estimates in the three surveillance scenarios.

**Conclusions:**

Differences between the assays can be primarily attributed to the calibrators supplied by the manufacturers. Performance for surveillance was extremely similar, although different thresholds were optimal (i.e. produced the lowest variance of incidence estimates) and at any given ODn threshold, different estimates of MDRI and FRR were obtained. The two assays cannot be treated as interchangeable: assay and algorithm-specific performance characteristic estimates must be used for survey planning and incidence estimation.

## Background

HIV incidence is a more sensitive indicator of epidemiological trends and intervention impact than prevalence; however, it is harder to estimate. Longitudinal follow-up of HIV-negative cohorts is considered the gold standard for estimating incidence, but is expensive, time-consuming, logistically challenging and results are difficult to generalize to the population level [1]. The field of HIV surveillance has therefore invested significant effort in developing methods for estimating incidence using cross-sectional surveys, notably by using laboratory assays to ascertain ‘recent’ infection [2–6]. The most widely-used ‘incidence assay’ is the Limiting Antigen Avidity EIA (LAg Assay), developed by the US Centers for Disease Control and Prevention (CDC) [7]. It has been used in major population-level HIV surveillance efforts, including the US Government-supported Population-based HIV Impact Assessment (PHIA) surveys conducted in several high-burden countries [8, 9] and the South African National HIV Prevalence, Incidence, Behaviour and Communication Survey [10].

Two major manufacturers supply versions of the assay: Maxim Biomedical (Bethesda, MD) and Sedia Biosciences Corporation (Portland, OR), with both manufacturers currently utilizing multisubtype HIV-1 recombinant antigen supplied by the CDC. A third manufacturer, Beijing King Hawk Pharmaceutical Co. (Beijing, PRC), has recently entered the market, but without US CDC involvement [11].

The performance of a test for recent infection is reflected in two key characteristics: the mean duration of recent infection (MDRI) and the false-recent rate (FRR). MDRI is the average time an individual spends in the ‘recent infection’ state as defined by a biomarker or set of biomarkers, having been infected for less than a cutoff time denoted *T*. FRR (also referred to as the false-recent ratio and the proportion falsely recent) is the proportion of individuals infected for longer than *T* who nevertheless exhibit the ‘recent’ biomarker. The accuracy of cross-sectional incidence estimates depends on applying correct context-specific test property estimates, while the precision of incidence estimates is sensitive to both MDRI and FRR. Choice of a recency discrimination threshold on a biomarker therefore implies a tradeoff between these two properties—maximizing MDRI and minimizing FRR [12].

Users of the Maxim and Sedia assays have generally assumed that they perform identically, with users of the Maxim assay using test property estimates published for the Sedia assay [13]. A recent comparison of the assays, based on 1,410 antiretroviral treatment (ART)-naïve HIV-1 subtype C-infected specimens, found substantially lower normalized optical densities (attributed to differences in calibrators) and consequently a longer MDRI (at the ‘standard’ recency discrimination threshold) for the Maxim assay [14].

In this study we confirm the systematic differences in reactivity between the Maxim and Sedia assays, while additionally conducting the first large-scale evaluation of the Maxim assay, including a comparative assessment of performance relative to the Sedia LAg assay, previously evaluated on the same specimen panel by the Consortium for the Evaluation and Performance of HIV Incidence Assays (CEPHIA) [15, 16]. Unlike the the study by Schlusser et al. published in 2017, we were able to robustly estimate performance characteristics, thanks to the use of a blinded 2,500-specimen panel (the CEPHIA Evaluation Panel), designed for this purpose and which represents a diversity of HIV-1 subtypes, observations at a range of times post-infection, and the inclusion of both treatment-naïve and virally suppressed specimens.

We have further developed a sophisticated approach for assessing real-world performance by estimating context-specific MDRIs and FRRs, taking into account study design and epidemiological factors such as the HIV-1 subtype mix in the infected population, the rate of viral suppression (primarily associated with ART, but also resulting from the small number of individuals capable of naturally suppressing viral replication in the absence of ART, known as ‘elite controllers’) and the distribution of times-since-infection in the surveyed population (see [17]). The unique features of our specimen panel and these methods that were not available in previous studies allow us to present the first true performance comparison of the two assays for HIV incidence surveillance.

## Methods

### The CEPHIA Evaluation Panel

The CEPHIA specimen repository houses more than 29,000 unique specimens from over 3,000 HIV-1-positive subjects. The Evaluation Panel (EP) consists of 2,500 plasma specimens [15, 16] that were obtained from 928 unique subjects, spanning a wide range of times since infection, and most infected with HIV-1 subtype B (57%), C (27%), A1 (10%), and D (5%). The panel contains 25 blinded replicates of 3 control specimens with antibody reactivity characteristic of recent, intermediate, and long-standing infection for reproducibility assessment, and specimens from ART-suppressed and naturally suppressed subjects to assess the impact of viral suppression on FRR.

The majority of subjects (68%) had sufficient clinical background data to produce Estimated Dates of Detectable Infection (EDDIs). These are infection time ‘point estimates’ accompanied by plausible intervals of first detectability, obtained by systematically interpreting diverse diagnostic testing histories according to the method previously described [18]. A subject’s EDDI represents the date on which a viral load assay with a 50% limit of detection of 1 RNA copy/mL would be expected to first detect the infection, and consequently MDRI estimates are ‘anchored’ to this reference test.

All patient-level data were fully anonymized before inclusion in this study, and the requirement for informed consent was waived; the University of California, San Francisco Human Research Protection Program and IRB (formerly CHR, #10-02365) approved the study procedures via expedited review for research involving materials previously collected for research purposes. All materials were collected under IRB-approved protocols.

### Laboratory procedures

The CEPHIA EP was tested with the Maxim and Sedia™ HIV-1 Limiting Antigen Avidity EIA (LAg) assays, according to their respective product inserts [19, 20]. Both assays are microtitre-based with the solid phase of the microtitre plate coated with a multi-subtype recombinant HIV-1 antigen. This antigen is coated in a limiting concentration to prevent crosslinking of antibody binding, making it easier to remove weakly-bound antibody. Specimen dilutions are incubated for 60 minutes and then a disassociation buffer is added for 15 minutes to remove any weakly-bound antibody. A goat anti-human, horseradish peroxidase (HRP)-conjugated IgG is added and this binds to any remaining IgG; a tetramethylbenzidine substrate is added and a colour is generated which is proportionate to the amount of HRP. An optical density (OD) is measured for each sample and this is normalized by use of a calibrator specimen. On each plate, the calibrator is tested in triplicate, with the median of the three ODs used to normalize specimen readings, producing normalized optical density (ODn) measurements.

The procedures for both assays are essentially the same, and both manufacturers source the recombinant antigen from the CDC as part of their licensing agreement. However, other components of the assay, such as the type of plates used, the control and calibrator materials, etc., were sourced or produced by the individual manufacturers. The testing procedure for both assays requires that specimens producing an initial ‘screening’ OD of ≤2.0 be subjected to triplicate ‘confirmatory’ testing. The median ODn of the triplicate results then serves as the final result [19, 20]. In the Maxim evaluation, a small number of specimens erroneously did not receive the triplicate confirmatory testing (12 out of 952), but a simulation investigation showed that this minor protocol deviation did not substantially affect results. It is further recommended that specimens producing an initial ODn ≤0.4 be subjected to serological confirmation of HIV infection.

Laboratory technicians were blinded to specimen background data during testing, which for each of the assays was completed in batches over a one month period using kits procured from the relevant manufacturer at the same time.

### Evaluation of assay performance for HIV incidence surveillance

We evaluated the performance of recent infection testing algorithms (RITAs)—so called when multiple criteria are used to define a ‘recent’ infection—based on the Maxim and Sedia LAg assays. A RITA typically consists of a screening assay to ascertain HIV infection followed by a single immunoassay (e.g. Maxim LAg or Sedia LAg) as primary marker of ‘recent infection’, followed by a quantitative viral load and sometimes antiretroviral (ARV) drug exposure testing. The addition of viral load and ARV exposure criteria to RITAs are critical in populations with significant ART coverage, since immunoassays tend to produce very high false-recent rates in virally suppressed subjects.

As noted above, we defined the performance of a RITA for incidence surveillance as the precision of the incidence estimates obtained. In order to evaluate performance, we therefore specified three hypothetical surveillance scenarios defined by HIV-1 prevalence, incidence, the distribution of HIV-1 subtypes in the population, ART coverage and viral suppression rates, as well as a survey sample size. Scenario A represents an epidemic similar to that of South Africa, Scenario B is similar to the Kenyan epidemic and Scenario C represents a concentrated (key population) epidemic. The assumptions defining each of the scenarios are summarised in Table 1. For each scenario and RITA (representing a threshold combination), we estimated context-specific MDRIs and FRRs.

**Table 1:** Hypothetical epidemiological scenarios for evaluating RITA performance in HIV incidence surveillance.

Parameter	Scenario A *South Africa-like*	Scenario B *Kenya-like*	Scenario C *Concentrated*
HIV-1 Subtype distribution:			
*Subtype A*	0%	70%	0%
*Subtype B*	0%	0%	100%
*Subtype C*	100%	5%	0%
*Subtype D*	0%	25%	0%
Prevalence: PE (SE)	18.9% (1.12%)	5.4% (0.36%)	15.0% (1.00%)
Incidence: PE (SE) *cases/100PY*	0.990 (0.0004)	0.146 (0.039)	0.5 (0.050)
ART coverage: PE (SE)	56% (5.6%)	64% (6.4%)	90% (9.0%)
Viral suppression rate: PE (SE)	82% (8.2%)	81% (8.1%)	90% (9.0%)
Surevey sample size:	35,000	14,000	5,000

**PE:** Point estimate. **SE:** Standard error. **PY:** Person-years.

We considered RITAs in which HIV-1 infection is detected by a fourth-generation (antigen-antibody ‘combo’) chemiluminescent assay and ‘recent infection’ is defined using a combination of biomarkers:

- an ODn below a specific threshold (on either the Maxim or Sedia LAg assay);
- a viral load above a specific threshold;
- a negative result on a test for the presence of ARVs.

We investigated a wide range of ODn threholds. In practice, the most commonly-used viral load threshold is >1000c/mL, and this threshold was used for the primary results presented here. Alternative viral load thresholds of 75, 400, 1,000, and 5,000c/mL were investigated and are reported in the supplemental material. The assumption that testing for ARVs accurately classifies all treated subjects as long-term infections is relaxed in sensitivity analyses reported in the supplemental material. Context-specific MDRI and FRRs were obtained for each RITA under each scenario in order to evaluate the precision of incidence estimates expected. The precision of incidence estimates is highly sensitive to FRR, and in most cases values above about 1% result in poor precision.

### Statistical analysis

The definitions of MDRI and FRR require the specification of a cut-off time *T* (set at 2 years in this study). When biomarker results suggesting ‘recent infection’ are obtained from individuals infected for longer than *T*, these are defined as ‘falsely recent’ [21].

We estimated MDRI by fitting binomial regression models for the probability of exhibiting the recent marker as a function of time since detectable infection *t* using data from subjects infected for less than 800 days, and integrated this function, *P*_*R*_(*t*), from 0 to *T* to obtain the average time individuals spend exhibiting the ‘recent’ marker. Confidence intervals were approximated by means of subject-level bootstrap resampling (10,000 iterations). MDRI may be sensitive to HIV-1 subtype, which affects post-infection antibody dynamics [15–17], so context-specific MDRIs were estimated by obtaining weighted averages of subtype-specific MDRIs were utilized in surveillance scenarios.

Naïve FRR estimates (i.e., not adated to epidemiological context), and their confidence intervals, were obtained by estimating the binomial probability that an untreated individual would produce a ‘recent’ result on the RITA when infected for longer than *T*. To obtain context-specific FRR estimates, we obtained a weighted average of FRR estimates for the treated and untreated HIV-positive subpopulations, weighted according to treatment coverage. To estimate FRR in untreated individuals we fitted *P*_*R*_(*t*) for all times post-infection and weighted that function by the probability density function of times-since-infection in the untreated population, parameterized as a Weibull survival function whose shape and scale parameters were chosen to produce a weighting function consistent with recent incidence, prevalence and treatment coverage. We estimated the FRR in treated individuals as the binomial probability that a treated individual infected for longer than *T* produces a recent result.

We used *inctools* R package [22] and extensions thereto [23] for MDRI and FRR estimation, as well as to obtain the expected relative standard error (RSE) on incidence estimates (i.e. the standard error as a proportion of the point estimate), given RITA properties, hypothesized incidence, prevalence and survey sample size. We demonstrate the recommended procedure by taking into account uncertainty in both the calibration data and contextual parameters specified in the three scenarios.

FRR depends strongly on context, since viral suppression, either as a result of ART or spontaneous viral suppression, frequently results in partial seroreversion which leads to the production of falsely-recent results on serological markers. Inclusion of viral load in a RITA (i.e. viral load less than some threshold results in classification as long-term infection, irrespective of ODn result) ameliorates the impact of viral suppression. In practice, a viral load threshold of >1,000c/mL is frequently used, especially when dried blood spot (DBS) specimens are collected for recency ascertainment. To obtain context-specific FRR estimates, denoted *ϵ*_*T*_, we estimated FRR in untreated individuals by fitting *P*_*R*_(*t*) for all times post-infection and weighted it by the probability density function of times-since-infection amongst the untreated population *ρ*(*t*), the latter parameterized as a Weibull survival function whose shape and scale parameters were chosen to produce a weighting function consistent with prevalence and treatment coverage, and normalized to recent incidence. We estimated the FRR in treated individuals, *P*_*R*|*tx*_, as the binomial probability that a treated individual infected for longer than T tests recent. We then obtain a weighted FRR estimate as shown in Eq 1 below.

$$\begin{array}{c} \epsilon_{T}=c\cdot P_{R|tx}+\left(1-c\right)\cdot \int_{T}^{\inf}\rho \left(t\right)P_{R}\left(t\right)\;dt \end{array}$$ (1)

where *c* is the treatment coverage,

$$\begin{array}{c} \rho \left(t\right)=\frac{f(t)}{\int_{T}^{\inf}f\left(t\right)\;dt} \end{array}$$ (2)

and

$$\begin{array}{c} f\left(t\right)=\exp(-{\left(\frac{t}{\alpha }\right)}^{\beta }) \end{array}$$ (3)

with *α* and *β* in Eq 3 the Weibull scale and shape parameters, respectively. This approach was previously described in [17] and [24].

While we have declared hypothetical scenarios in which epidemiological parameters are ‘known’, we demonstrate the procedure that would be recommended in real-world settings by taking into account uncertainty in these parameters. To evaluate reproducibility of FRR estimates, we bootstrapped (30,000 iterations) both the calibration data and contextual parameters, the latter drawn from truncated normal distributions with means and standard deviations as defined for the scenarios above.

The extensions to the *inctools* R package [22] that implement these methods are available publicly [23].

## Results

### Calibrators and reproducibility on replicate control specimens

As reported in Table 2, the mean OD for all Maxim calibrators was 0.75 and for Sedia was 0.65, a difference in means of 0.107 (95% CI: 0.090,0.123, p-value from Welch two-sample t-test < 0.001). When restricted to only the calibrators used for normalization—i.e., the median value of the three ODs obtained from triplicate testing on each plate—the coefficients of variation (CVs) of Maxim and Sedia calibrators were 9.3% and 14.2%, respectively. The distributions of calibrator reactivity are shown in S1 Fig.

**Table 2:** Calibrator reactivity and reproducibility of results assessed by repeat testing.

Specimen	Maxim					Sedia				
	N	Mean OD	CV OD (%)	Mean ODn	CV ODn (%)	N	Mean OD	CV OD (%)	Mean ODn	CV ODn (%)
*Calibrators*										
All^a^	222	0.75	10.4	*N/A*	*N/A*	219	0.65	15.0	*N/A*	*N/A*
Median values^b^	74	0.75	9.3	*N/A*	*N/A*	73	0.64	14.2	*N/A*	*N/A*
*Kit-supplied control specimens*										
Acute (low)	222	0.36	10.5	0.49	8.0	219	0.35	16.2	0.55	14.0
Chronic (high)	222	1.37	8.6	1.83	7.9	219	1.31	10.4	2.06	12.7
*Blinded control specimens*										
BC-1	25	3.30	5.1	4.45	9.3	25	3.07	5.6	4.94	15.0
BC-2	25	3.04	5.9	4.08	8.9	25	2.83	6.2	4.5	13.2
BC-3	25	0.40	14.2	0.54	14.8	25	0.67	20.0	1.02	13.6

**^a^**Average over all calibrator values;

**^b^**Average over median calibrator values (one value per plate).

Reproducibility on the three blinded control specimens was similar, with CVs on OD and ODn (across 25 replicates) slightly higher for Sedia. The Maxim assay produced lower ODn values on average, and a much lower mean ODn on the low-reactivity specimen (labelled BC-3), of 0.54 vs. 1.02 on the Sedia assay. In accordance with the manufactures’ instructions for use, specimen BC-3 was subjected to triplicate confirmatory testing on both assays. The reported ODs were those obtained from the initial screening runs, and the mean and CV on ODn results were computed on the 25 final values.

### Performance on clinical specimens

**Fig 1:**
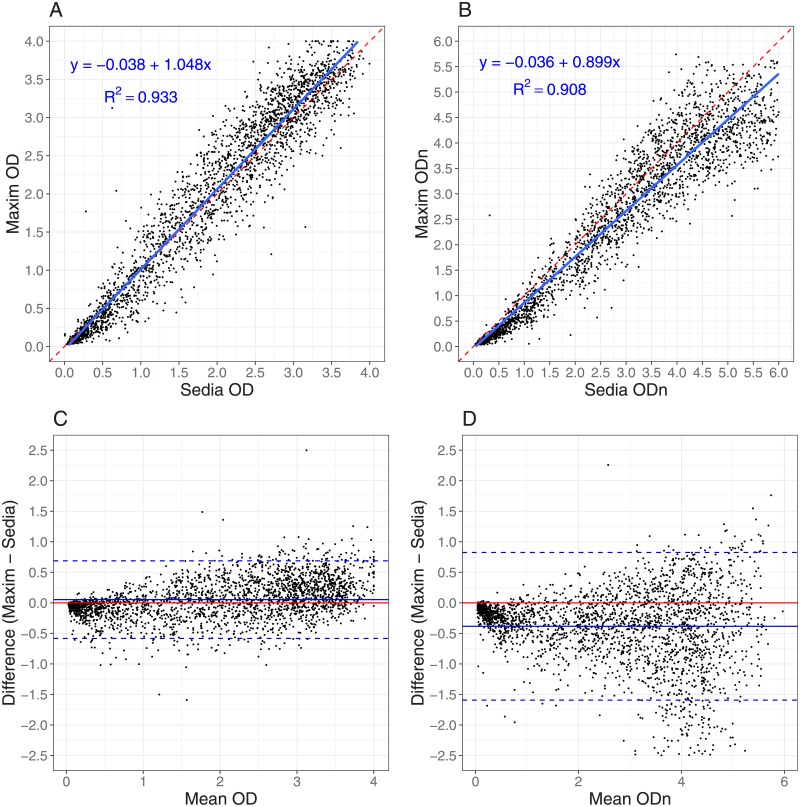
Maxim vs. Sedia OD and ODn measurements. **A:** Maxim vs. Sedia Optical Density (OD); **B:** Maxim vs. Sedia normalized Optical Density (ODn). The blue lines are linear regression fits and the red dashed lines show the diagonal (slope if the two assays produced equivalent results). **C:** Bland-Altman plot for Optical Density (OD); **D:** Bland-Altman plot for normalized Optical Density (ODn). The red lines represent zero bias, the blue solid lines the mean differences and the blue dashed lines the 95% lower and upper limits.

Fig 1 shows results of testing clinical specimens in the EP and the impact of the higher Maxim calibrator readings. ODn values in Fig 1B are concentrated below the diagonal line, especially at lower ODn values in the range of plausible recency discrimination thresholds. In fact, correlation was stronger for non-normalized OD readings than for normalized ODn readings. The slope for OD in Fig 1A is closer to unity than the slope for ODn in Fig 1B, which also shows poorer correlation. The linear regression slopes were statistically significantly different (*p* < 0.0001). The Bland-Altman plots in Fig 1C and 1D show that the Maxim assay tends to produce lower OD readings than the Sedia assay on the low end of the dynamic range, and higher readings at the top end. When the calibrators are used to normalize, Maxim ODn values exhibit clear downward bias throughout the dynamic range.

MDRI was estimated using treatment-naïve, non-elite controller subjects, with EDDI intervals ≤120 days. Using ODn ≤1.5, the MDRI for Maxim, without using a supplemental viral load, was 248 days (95% CI: 224,274), while the MDRI for Sedia was 215 days (95% CI: 192,241), resulting in a difference estimate of 32.7 days (95% CI: 22.9,42.8). Applying a supplemental viral load threshold of >1,000c/mL resulted in MDRI estimates of 201 days (95% CI: 180,224) and 171 days (95% CI: 152,191), respectively and a difference of 30.9 days (95% CI: 21.7,40.7).

Table 3 shows MDRI estimates for all subtypes, and by subtype (B, C, D and A1), for a range of ODn thresholds in combination with a viral load threshold (>1,000c/mL). We did not observe statistically significant differences between subtype-specific MDRI estimates and the estimates for all other subtypes combined (using a two-sample *Z*-test) for either assay at any ODn threshold. MDRI estimates for a wider range of ODn and viral load thresholds are reported in S1 Table.

**Table 3:** MDRI estimates for Maxim and Sedia LAg assays by HIV-1 subtype and ODn threshold, using supplemental viral load threshold of >1,000c/mL.

HIV Subtype	ODn≤	Maxim		Sedia	
		MDRI (95% CI)	p-value*	MDRI (95% CI)	p-value*
All	1.0	156 (139,176)	*N/A*	122 (106,138)	*N/A*
All	1.5	201 (180,223)	*N/A*	171 (152,191)	*N/A*
All	2.0	244 (220,268)	*N/A*	204 (183,227)	*N/A*
All	2.5	321 (294,350)	*N/A*	278 (252,305)	*N/A*
B	1.0	154 (119,203)	0.907	127 (91,175)	0.788
B	1.5	203 (162,255)	0.895	176 (132,226)	0.871
B	2.0	240 (191,295)	0.969	204 (160,257)	0.949
B	2.5	299 (245,357)	0.474	250 (201,307)	0.307
C	1.0	151 (130,175)	0.586	112 (97,131)	0.222
C	1.5	197 (170,226)	0.708	162 (141,185)	0.357
C	2.0	239 (207,272)	0.728	197 (170,225)	0.528
C	2.5	323 (285,363)	0.943	283 (245,321)	0.809
D	1.0	192 (109,292)	0.406	166 (86,262)	0.263
D	1.5	223 (140,321)	0.617	209 (126,307)	0.375
D	2.0	250 (164,350)	0.901	241 (152,347)	0.403
D	2.5	298 (203,406)	0.597	281 (186,391)	0.979
A1	1.0	182 (133,240)	0.340	147 (107,192)	0.240
A1	1.5	203 (148,265)	0.914	186 (137,245)	0.555
A1	2.0	261 (198,332)	0.536	205 (150,268)	0.950
A1	2.5	369 (299,435)	0.127	323 (258,386)	0.151

*To obtain these p-values we compare HIV-1 subtype-specific MDRI with the MDRI for all other subtypes, at the relevant ODn threshold, using a two-sided Z-test.

While naïvely-estimated FRRs at a given threshold were not identical between the Maxim and Sedia assays, the differences were not statistically significant. The FRRs in ART-naïve subjects (without using viral load) were 3.26% and 2.17% for Maxim and Sedia, respectively, at ODn ≤1.5, and 1.69% and 1.12%, respectively when using viral load >1000c/mL. These estimates are shown in S1 Table. Among treated subjects FRRs were extremely high when the RITA did not include a viral load threshold. In early-treated subjects (time from infection to treatment initiation ≤6 months), the FRRs for Maxim and Sedia were 98% and 96%, respectively, and in later-treated subjects (time from infection to treatment initiation >6 months), FRRs were 38% vs. 33%, respectively. Using a supplemental viral load threshold reduced these FRRs to 0, given that all treated subjects in the EP were virally suppressed.

### Performance in surveillance

**Fig 2:**
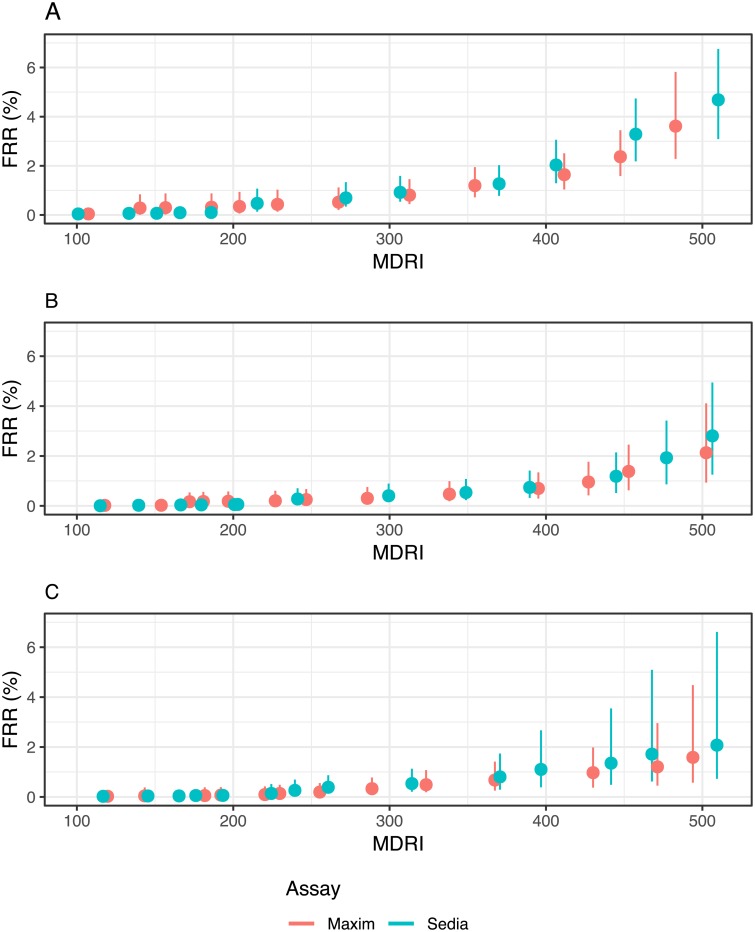
Context-specific false-recent rate (FRR) against MDRI in three demonstrative surveillance scenarios. **A:** Scenario similar to South African epidemic. **B:** Scenario similar to Kenyan epidemic. **C:** Concentrated epidemic scenario. A supplementary viral load threshold of >1,000c/mL is used throughout. We assume ARV exposure testing classifies all treated individuals as long-term. This assumption is relaxed in S4 Fig.

**Fig 3:**
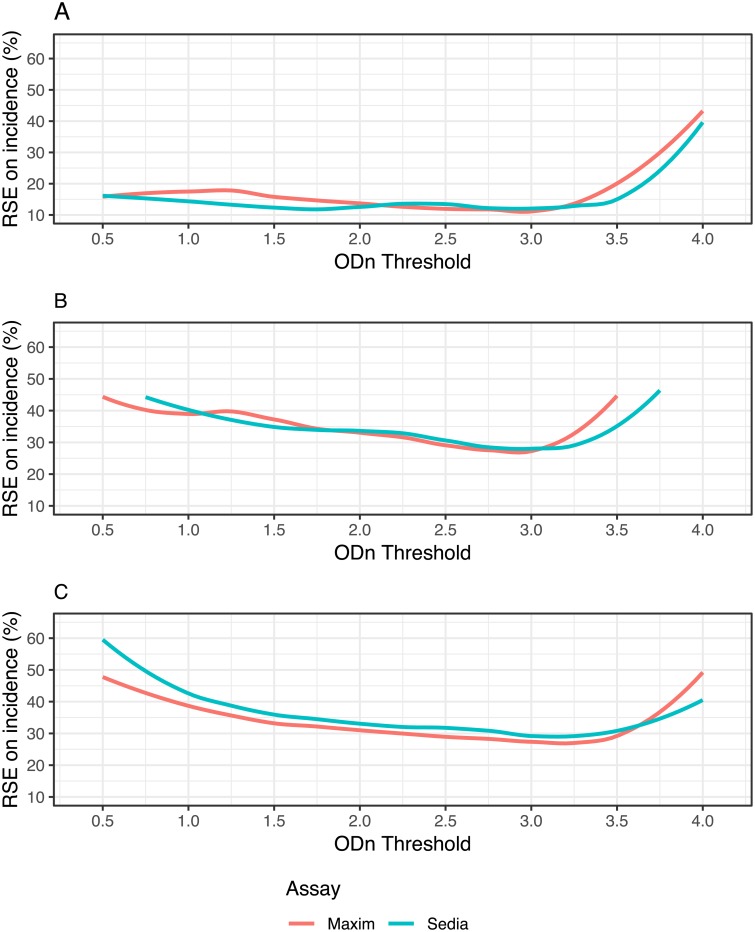
Relative standard error (RSE) of incidence estimate against ODn threshold in three demonstrative surveillance scenarios. **A:** Scenario similar to South African epidemic. **B:** Scenario similar to Kenyan epidemic. **C**. Concentrated epidemic scenario. A supplementary viral load threshold of >1,000c/mL is used throughout. We assume ARV exposure testing classifies all treated individuals as long-term. This assumption is relaxed in S5 Fig.

The performance of the two assays in the three hypothetical surveillance scenarios defined earlier are summarised in Figs 2 and 3.

Fig 2 shows context-specific FRR against context-specifc MDRI, for RITAs that included a viral load threshold of >1,000c/mL and where we assume that ART exposure testing reduces false recency in treated subjects to zero. Note that the MDRI values on the x-axis encode different ODn thresholds for the two assays. This figure visualises the tradeoff between MDRI and FRR as the ODn threshold is increased, under the assumptions of the three scenarios. The FRR rises at slightly lower MDRIs for Sedia-based RITAs than for Maxim-based RITAs, in all three scenarios. To maintain FRRs below 2%, both assays require a choice of ODn threshold that produces maximal MDRIs of about 400 to 450 days. In the supplemental material, we show context-adapted FRRs against ODn thresholds (S2 Fig) and we relax the assumption that ART exposure testing performs perfectly (S3 Fig). S4 Fig shows context-specific FRR against MDRI under the alternative assumption of imperfect ART exposure testing.

Fig 3 shows the precision of the incidence estimate attained for a range of ODn thresholds in combination with a viral load threshold of >1,000c/mL. At each ODn threshold, assay-specific context-adapted MDRIs and FRRs were computed for use in the incidence calculation. Context-specific MDRI and FRR estimates, and RSEs on incidence estimates, are reported in S2 Table. In the South Africa-like scenario,Fig 3A, the lowest value of RSE on incidence attained with the Maxim-based algorithm was 11.7% at ODn ≤3.0, and with the Sedia-based algorithm was 12.0% at the same ODn threshold. In the Kenya-like scenario, Fig 3B, the minimal RSE for Maxim was 27.2%, achieved at ODn ≤2.75, and for Sedia was 28.2% at ODn ≤3.0. In the North American key population-like scenario, Fig 3C, the lowest RSE for Maxim was 26.9% at ODn ≤3.25 and for Sedia was 28.9% at ODn ≤3.0. These nominally optimal thresholds were slightly different under the alternative assumption shown in S5 Fig.

## Discussion

The Maxim and Sedia LAg assays produce meaningfully different ODn results on the same specimens, largely as a result of higher readings obtained from the Maxim-supplied kit calibrators, and consequently, at any given ODn threshold, RITAs based on the two assays have different MDRIs and FRRs. It is inappropriate to utilize published MDRI and FRR estimates for one assay in survey planning and incidence estimation where the other assay is being used, or to switch from one assay to the other within a study.

It is possible to derive an approximate conversion factor (of 1.172) between ODn values of the two assays from the slopes of the regression curves shown in Fig 1A and 1B. It has further been suggested that a threshold of 1.5 on Maxim is equivalent to a threshold of 2.0 on Sedia, based on testing of a set of specimens, with reactivity spanning the dynamic range, with both assays (personal communication, B. Parekh). Our analysis does indeed show that these thresholds yield very similar MDRIs when used alone (248 days vs. 254 days), but the FRRs are also different. Applying a conversion factor to the Sedia results of repeat-tested specimens does not perfectly predict the Maxim ODn values obtained, and a preferable approach is therefore to use appropriately-estimated MDRIs and FRRs for any given RITA based on either assay.

Our reproducibility analyses show little benefit to the normalization procedure, with both the Maxim and Sedia assays showing greater variability in ODn values than in the raw optical densities on blinded replicate specimens subjected to repeat testing. Further, the correlation between Maxim and Sedia ODs was greater than between ODn measurements on the same specimens. However, at the time of each of these evaluations, kits and reagents were sourced at the same time, kits were from a small number of lots, and operators were highly experienced with assays. The purpose of the calibrators and normalization procedure is to reduce lot-to-lot variability and ensure stability of results over time and between manufacturers and laboratories. This goal requires that calibrators be highly consistent over time and between manufacturers, which is not currently the case. The NIAID-supported External Quality Assurance Program Oversight Laboratory (EQAPOL) LAG program found similar differences in calibrator reactivity and average ODn values between the two assays (Keating et al., forthcoming). External quality assurance is critical for ensuring consistency between laboratories and kit manufacturers.

It should be noted that our evaluation of both assays was restricted to plasma specimens. Both manufacturers also produce kits for use with dried blood spot eluates, and it has been shown that specimen type further impacts performance [25].

We did not observe any statistically significant subtype effects on MDRI, although point estimates differed substantially, especially with specimens from subtype D-infected subjects compared to subtypes B and C (Table 3). With a larger dataset and more precise MDRI estimates, subtype differences may be visible.

Despite the systematic differences in calibrator readings and consequently in the ODn values obtained, performance of the two assays for incidence surveillance was virtually indistinguishable—as long as appropriate assay- and context-specific MDRI and FRR estimates were used. As a result, different ODn thresholds were nominally optimal (i.e. produced the lowest variance on the incidence estimate). In all three hypothetical surveillance scenarios, ODn thresholds between about 1.5 and 3.25 (in combination with viral load), produced the best precision. It is critical, however, that appropriate MDRI and FRR estimates be used for the assay, recency discrimination threshold and other RITA components chosen in order to obtain accurate incidence estimates. Since there was no clear performance advantage to either assay in any of the scenarios, we do not recommend the choice of one over the other. However, RITAs based on either assay should be optimized in order to maximize the precision of incidence estimates, by choosing appropriate thresholds. RITAs based on both the Maxim and Sedia LAg perform well compared to those based on other assays for identifying recent HIV-1 infection [15–17].

It should also be noted that the triplicate ‘confirmatory’ testing protocol mandates confirmatory testing when an initial ODn result is below 2.0, which may be problematic for RITAs that use ODn thresholds above the ‘standard’ threshold of 1.5. It would also be a different subset of specimens reflexed to confirmatory testing on the two assays. Consideration should be given to a modified testing protocol in which confirmatory testing is performed on a larger subset of (or even all) specimens.

A limitation of this study is that we did not have access to specimens from virally unsuppressed treated subjects, and we are therefore unable to rigorously estimate FRR in this group, which may be substantial in many surveillance settings [26]. We urge survey planners and analysts to conduct sensitivity analyses with respect to FRR when utilising either assay in cross-sectional incidence estimation.

Differences in ODn measurements between the Maxim and Sedia LAg assays on the same specimens largely resulted from differences in the reactivity of calibrators supplied by the manufacturers. This resulted in systematically lower ODn measurements on the Maxim assay than on the Sedia assay, and consequently longer MDRIs and larger FRRs at any given ODn recency discrimination threshold. While performance for surveillance purposes was extremely similar, different thresholds were optimal and different values of MDRI and FRR were appropriate for use in survey planning and incidence estimation. The two assays cannot be treated as interchangeable, should not be mixed within one study, and care should be taken when interpreting and comparing results. We summarize our recommendations based on this comparative evaluation in Table 4.

**Table 4:** Summary recommendations for use of the Maxim and Sedia LAg assays.

	Issue	Recommendation
Laboratory methods:	Assay procedures are similar but not identical.	Testing laboratories should ensure full compliance with manufacturer’s instructions for use, especially if both manufacturers’ assays are used in one laboratory.
Quality assurance:	Lot-to-lot variation and differences in laboratory staff proficiency may further reduce reproducibility of results.	Continuous quality assurance should be practiced, including by ensuring laboratory staff proficiency, by regularly running well-characterized quality assurance specimens (recent, longterm and negative) and by monitoring the reactivity of kit-supplied specimens (controls and calibrators) over time. Participation in an external quality assurance programme like EQAPOL [27] is recommended.
Software:	Although data capture and analysis software are similar, interpretive criteria for specific components differ.	The data analysis software is specific to each assay and laboratories should use the software supplied by the manufacturer.
Conversion:	Although it is possible to compute an approximate conversion factor, this does not perfectly predict equivalent ODn values.	Rather than converting results, appropriately-derived MDRI and FRR estimates should be utilized for each assay. The same ODn thresholds may not be optimal.
Descriptive title:	The names ‘HIV-1 Limiting Antigen Avidity EIA’ or ‘LAg assay’ do not distinguish between the two assays.	Users should clearly identify the manufacturer of the kits used, as well as specimen type, in all publications and reports.
Assay performance:	Despite differences in calibrator reactivity, and consequently in ODn values obtained on the same specimens, performance of the two assays for surveillance purposes was virtually indistinguishable.	Both manufacturers’ assays are suitable for use, but they should not be mixed within studies, appropriate performance characteristic estimates must be used and care should be taken when comparing results.

## Supporting information

S1 Table MDRI and FRR estimates in ART-naïve subjects for a range of ODn and viral load thresholds. (PDF)

S2 Table Context-specific MDRI and FRR estimates from the three demonstrative surveillance scenarios under different assumptions about impact of ARV exposure testing on FRR. (PDF)

S1 Fig Density plot of Maxim and Sedia calibrator ODs. (EPS)

S2 Fig Context-specific FRR vs. ODn threshold in three demonstrative surveillance scenarios (assuming perfect ARV exposure testing). **A:** Scenario similar to South African epidemic. **B:** Scenario similar to Kenyan epidemic. **C:** Concentrated epidemic scenario. A supplementary viral load threshold of >1,000c/mL is used throughout. We assume ARV exposure testing classifies all treated individuals as long-term.
(EPS)

S3 Fig Context-specific FRR vs. ODn threshold in three demonstrative surveillance scenarios (assuming imperfect ARV exposure testing). **A:** Scenario similar to South African epidemic. **B:** Scenario similar to Kenyan epidemic. **C:** Concentrated epidemic scenario. A supplementary viral load threshold of >1,000c/mL is used throughout. We assume ARV exposure testing reduces false recency in treated individuals to 10% of that attained when no supplemental viral load threshold is utilized.
(EPS)

S4 Fig Context-specific false-recent rate (FRR) against MDRI in three demonstrative surveillance scenarios (assuming imperfect ARV testing). **A:** Scenario similar to South African epidemic. **B:** Scenario similar to Kenyan epidemic. **C:** Concentrated epidemic scenario. A supplementary viral load threshold of >1,000c/mL is used throughout. We assume ARV exposure testing reduces false recency in treated individuals to 10% of that attained when no supplemental viral load threshold is utilized.
(EPS)

S5 Fig Relative standard error (RSE) of incidence estimate against ODn threshold in three demonstrative surveillance scenarios (assuming imperfect ARV exposure testing). **A:** Scenario similar to South African epidemic. **B:** Scenario similar to Kenyan epidemic. **C:** Concentrated epidemic scenario. Assuming imperfect ARV exposure testing which reduces the FRR in virally unsuppressed treated individuals to 10% of FRR in suppressed individuals when no viral load threshold applied.
(EPS)

S1 Dataset Minimal dataset. CEPHIA Evaluation Panel dataset containing final ODn values for each assay, excluding blinded control specimens.
(CSV)

S2 Dataset Blinded control specimens. Results from 25 replicates of each of three blinded control specimens included in the CEPHIA Evaluation Panel.
(CSV)
