# Supplementary material for: Performance comparison of the Maxim and Sedia Limiting Antigen Avidity assays for HIV incidence surveillance
Source: PLoS One. 2019 Jul 26;14(7):e0220345. doi: 10.1371/journal.pone.0220345 (PMC6660077; doi:10.1371/journal.pone.0220345)
Supplement: S1 Table — (PDF) [file pone.0220345.s001.pdf]

S1 Table: MDRI and FRR* estimates in ART-naïve subjects for a range of ODn and viral load thresholds

ODn	VL	Maxim		Sedia	
≤	¿	MDRI (95% CI)	FRR (95% CI)	MDRI (95% CI)	FRR (95% CI)
0.50	None	129 (113,146)	1.6% (0.3,4.7)	91 (79,105)	0.5% (0.0,3.0)
0.75	None	164 (144,184)	1.6% (0.3,4.7)	127 (111,145)	1.6% (0.3,4.7)
1.00	None	197 (176,220)	2.2% (0.6,5.5)	158 (138,179)	1.6% (0.3,4.7)
1.25	None	224 (200,248)	2.7% (0.9,6.2)	194 (172,217)	2.2% (0.6,5.5)
1.50	None	248 (224,274)	3.3% (1.2,7.0)	215 (192,240)	2.2% (0.6,5.5)
1.75	None	277 (251,304)	3.8% (1.5,7.7)	237 (213,263)	4.4% (1.9,8.4)
2.00	None	297 (270,324)	6.5% (3.4,11.1)	254 (228,280)	4.4% (1.9,8.4)
2.25	None	338 (311,365)	7.6% (4.2,12.4)	292 (265,319)	6.0% (3.0,10.4)
2.50	None	382 (354,410)	11.4% (7.2,16.9)	333 (305,362)	9.8% (5.9,15.0)
2.75	None	423 (394,453)	16.9% (11.7,23.1)	373 (344,401)	13.0% (8.5,18.8)
3.00	None	471 (442,500)	20.1% (14.6,26.6)	427 (398,457)	16.3% (11.3,22.5)
0.50	0**	129 (113,147)	1.7% (0.4,4.9)	91 (79,105)	0.6% (0.0,3.1)
0.50	75	118 (104,132)	1.1% (0.1,4.0)	83 (73,94)	0.6% (0.0,3.1)
0.50	400	108 (96,122)	0.6% (0.0,3.1)	78 (68,88)	0.0% (0.0,2.1)
0.50	1,000	102 (90,114)	0.6% (0.0,3.1)	73 (64,82)	0.0% (0.0,2.1)
0.50	5,000	86 (75,97)	0.6% (0.0,3.1)	63 (54,72)	0.0% (0.0,2.1)
0.75	0**	165 (145,186)	1.7% (0.4,4.9)	127 (110,146)	1.7% (0.4,4.9)
0.75	75	151 (133,169)	1.1% (0.1,4.0)	116 (102,132)	0.6% (0.0,3.1)
0.75	400	138 (121,155)	0.6% (0.0,3.1)	106 (93,120)	0.6% (0.0,3.1)
0.75	1,000	127 (112,144)	0.6% (0.0,3.1)	99 (86,112)	0.6% (0.0,3.1)
0.75	5,000	106 (92,122)	0.6% (0.0,3.1)	83 (71,96)	0.6% (0.0,3.1)
1.00	0**	199 (177,223)	2.3% (0.6,5.7)	159 (139,181)	1.7% (0.4,4.9)
1.00	75	183 (164,205)	1.1% (0.1,4.0)	145 (127,164)	1.1% (0.1,4.0)
1.00	400	168 (149,188)	1.1% (0.1,4.0)	130 (114,148)	0.6% (0.0,3.1)
1.00	1,000	156 (139,176)	1.1% (0.1,4.0)	122 (106,138)	0.6% (0.0,3.1)
1.00	5,000	126 (109,144)	1.1% (0.1,4.0)	101 (86,117)	0.6% (0.0,3.1)
1.25	0**	226 (202,251)	2.8% (0.9,6.4)	197 (174,221)	2.3% (0.6,5.7)
1.25	75	210 (188,233)	1.7% (0.4,4.9)	180 (161,201)	1.7% (0.4,4.9)
1.25	400	191 (171,212)	1.7% (0.4,4.9)	164 (146,184)	1.1% (0.1,4.0)
1.25	1,000	179 (160,199)	1.7% (0.4,4.9)	153 (135,172)	1.1% (0.1,4.0)
1.25	5,000	141 (123,160)	1.7% (0.4,4.9)	122 (106,140)	1.1% (0.1,4.0)
1.50	0**	250 (225,276)	3.4% (1.3,7.2)	218 (194,244)	2.3% (0.6,5.7)
1.50	75	233 (210,256)	1.7% (0.4,4.9)	202 (180,224)	1.7% (0.4,4.9)
1.50	400	214 (193,237)	1.7% (0.4,4.9)	183 (163,204)	1.1% (0.1,4.0)
1.50	1,000	201 (180,223)	1.7% (0.4,4.9)	171 (152,191)	1.1% (0.1,4.0)
1.50	5,000	158 (138,179)	1.7% (0.4,4.9)	135 (118,154)	1.1% (0.1,4.0)
1.75	0**	279 (252,306)	3.9% (1.6,7.9)	239 (213,266)	4.5% (2.0,8.7)
1.75	75	261 (236,286)	1.7% (0.4,4.9)	222 (199,245)	2.3% (0.6,5.7)
1.75	400	242 (219,266)	1.7% (0.4,4.9)	203 (182,225)	1.7% (0.4,4.9)
1.75	1,000	227 (206,251)	1.7% (0.4,4.9)	190 (170,211)	1.7% (0.4,4.9)
1.75	5,000	175 (155,198)	1.7% (0.4,4.9)	148 (129,168)	1.7% (0.4,4.9)
2.00	0**	299 (272,326)	6.7% (3.5,11.5)	254 (228,282)	4.5% (2.0,8.7)
2.00	75	281 (255,307)	5.1% (2.3,9.4)	236 (212,261)	2.8% (0.9,6.4)
2.00	400	261 (237,286)	3.9% (1.6,7.9)	217 (196,241)	1.7% (0.4,4.9)
2.00	1,000	244 (220,268)	3.9% (1.6,7.9)	204 (183,227)	1.7% (0.4,4.9)
2.00	5,000	188 (166,210)	3.9% (1.6,7.9)	160 (141,180)	1.7% (0.4,4.9)
2.25	0**	339 (312,367)	7.9% (4.4,12.8)	292 (265,320)	6.2% (3.1,10.8)
2.25	75	321 (295,348)	6.2% (3.1,10.8)	274 (249,300)	4.5% (2.0,8.7)
2.25	400	300 (276,326)	4.5% (2.0,8.7)	253 (230,278)	3.4% (1.3,7.2)

ODn	VL	Maxim		Sedia	
≤	¿	MDRI (95% CI)	FRR (95% CI)	MDRI (95% CI)	FRR (95% CI)
2.25	1,000	280 (255,306)	4.5% (2.0,8.7)	237 (214,261)	3.4% (1.3,7.2)
2.25	5,000	214 (190,239)	3.9% (1.6,7.9)	186 (164,209)	2.8% (0.9,6.4)
2.50	0**	383 (354,411)	11.2% (7.0,16.8)	335 (306,364)	10.1% (6.1,15.5)
2.50	75	364 (336,392)	9.6% (5.7,14.9)	316 (289,344)	8.4% (4.8,13.5)
2.50	400	343 (316,371)	7.9% (4.4,12.8)	296 (269,322)	6.7% (3.5,11.5)
2.50	1,000	321 (294,350)	7.9% (4.4,12.8)	278 (252,305)	6.7% (3.5,11.5)
2.50	5,000	252 (225,280)	6.2% (3.1,10.8)	220 (194,246)	5.1% (2.3,9.4)
2.75	0**	426 (397,455)	17.4% (12.2,23.8)	375 (346,405)	13.5% (8.8,19.4)
2.75	75	406 (377,434)	15.7% (10.7,21.9)	357 (328,386)	11.8% (7.5,17.5)
2.75	400	384 (355,413)	13.5% (8.8,19.4)	336 (308,364)	10.1% (6.1,15.5)
2.75	1,000	363 (334,393)	13.5% (8.8,19.4)	315 (287,343)	9.6% (5.7,14.9)
2.75	5,000	285 (256,315)	10.7% (6.6,16.2)	250 (223,279)	6.2% (3.1,10.8)
3.00	0**	475 (445,505)	20.8% (15.1,27.5)	431 (401,461)	16.9% (11.7,23.2)
3.00	75	455 (426,483)	19.1% (13.6,25.7)	412 (382,441)	15.2% (10.2,21.3)
3.00	400	430 (401,459)	16.9% (11.7,23.2)	391 (362,420)	13.5% (8.8,19.4)
3.00	1,000	408 (379,437)	16.9% (11.7,23.2)	369 (340,396)	13.5% (8.8,19.4)
3.00	5,000	326 (297,358)	12.9% (8.4,18.8)	296 (266,327)	9.6% (5.7,14.9)

*Naïvely-estimated FRR, i.e. not adapted to epidemiological context.

**No viral load threshold is applied, but analysis restricted to specimens that have viral load data available.
