# Supplementary material for: Performance comparison of the Maxim and Sedia Limiting Antigen Avidity assays for HIV incidence surveillance
Source: PLoS One. 2019 Jul 26;14(7):e0220345. doi: 10.1371/journal.pone.0220345 (PMC6660077; doi:10.1371/journal.pone.0220345)
Supplement: S2 Table — (PDF) [file pone.0220345.s002.pdf]

S2 Table: Context-specific MDRI and FRR estimates from the three demonstrative surveillance scenarios under different assumptions about impact of ARV exposure testing on FRR

				Assmuption 1*		Assumption 2**	
Assay	ODn	Scenario§	MDRI† (95% CI)	FRR (95% CI)	RSE	FRR (95% CI)	RSE
Maxim	0.5	A	88 (74,104)	0.0% (0.0,0.1)	16.7%	0.6% (0.1,2.4)	63.8%
Maxim	1.0	A	140 (119,165)	0.3% (0.0,0.8)	19.0%	0.9% (0.1,3.0)	46.8%
Maxim	1.5	A	186 (159,214)	0.3% (0.0,0.9)	15.6%	1.0% (0.2,3.3)	38.1%
Maxim	2.0	A	228 (196,261)	0.4% (0.1,1.0)	13.8%	1.2% (0.3,3.7)	34.2%
Maxim	2.5	A	313 (273,353)	0.8% (0.4,1.5)	11.8%	1.7% (0.7,4.4)	27.3%
Sedia	0.5	A	73 (61,85)	0.0% (0.0,0.0)	16.1%	0.5% (0.0,2.0)	63.5%
Sedia	1.0	A	101 (86,120)	0.0% (0.0,1.0)	14.7%	0.7% (0.1,2.6)	58.9%
Sedia	1.5	A	151 (130,174)	0.1% (0.0,0.2)	12.3%	0.8% (0.1,2.8)	42.1%
Sedia	2.0	A	186 (159,215)	0.1% (0.0,0.2)	11.7%	0.8% (0.1,3.0)	36.5%
Sedia	2.5	A	272 (234,311)	0.7% (0.3,1.3)	13.1%	1.5% (0.6,4.1)	30.0%
Maxim	0.5	B	88 (74,104)	0.0% (0.0,0.1)	16.7%	0.6% (0.1,2.4)	63.8%
Maxim	1.0	B	140 (119,165)	0.3% (0.0,0.8)	19.0%	0.9% (0.1,2.0)	46.8%
Maxim	1.5	B	186 (159,214)	0.3% (0.0,0.9)	15.6%	1.0% (0.2,3.3)	38.1%
Maxim	2.0	B	228 (196,261)	0.4% (0.1,1.0)	13.8%	1.2% (0.3,3.7)	34.2%
Maxim	2.5	B	313 (273,353)	0.8% (0.4,1.5)	11.8%	1.7% (0.7,4.4)	27.3%
Sedia	0.5	B	73 (61,85)	0.0% (0.0,0.0)	16.1%	0.5% (0,0.02)	63.5%
Sedia	1.0	B	101 (86,120)	0.0% (0.0,0.1)	14.7%	0.7% (0.1,2.6)	58.9%
Sedia	1.5	B	151 (130,174)	0.1% (0.0,0.2)	12.3%	0.8% (0.1,2.8)	42.1%
Sedia	2.0	B	186 (159,215)	0.1% (0.0,0.2)	11.7%	0.8% (0.1,3.0)	36.5%
Sedia	2.5	B	272 (234,311)	0.7% (0.3,1.3)	13.1%	1.5% (0.6,4.1)	29.9%
Maxim	0.5	C	88 (74,104)	0.0% (0.0,0.1)	16.7%	0.6% (0.1,2.4)	63.8%
Maxim	1.0	C	140 (119,165)	0.3% (0.0,0.8)	19.0%	0.9% (0.1,3.0)	46.8%
Maxim	1.5	C	186 (159,214)	0.3% (0.0,0.9)	15.6%	1.0% (0.2,3.3)	38.1%
Maxim	2.0	C	228 (196,261)	0.4% (0.1,1.0)	13.8%	1.2% (0.3,3.7)	34.2%
Maxim	2.5	C	313 (273,353)	0.8% (0.4,1.5)	11.8%	1.7% (0.7,4.4)	27.3%
Sedia	0.5	C	73 (61,85)	0.0% (0.0,0.0)	16.1%	0.5% (0.0,2.0)	63.5%
Sedia	1.0	C	101 (86,120)	0.0% (0.0,0.1)	14.7%	0.7% (0.1,2.6)	58.9%
Sedia	1.5	C	151 (130,174)	0.1% (0.0,0.2)	12.3%	0.8% (0.1,2.8)	42.0%
Sedia	2.0	C	186 (159,215)	0.1% (0.0,0.2)	11.7%	0.8% (0.1,3.0)	36.5%
Sedia	2.5	C	272 (234,311)	0.7% (0.3,1.3)	13.1%	1.5% (0.6,4.1)	29.9%

§Scenario **A:** South Africa-like epidemic; **B:** Kenya-like epidemic; **C:** Concentrated epidemic.

†Adjusted for subtype mix and screening assay

*ARV exposure testing classifies all treated subjects as long-term

**ARV exposure testing reduces FRR in treated subjects to 10% of FRR without viral load
